# Balance between a Higher Degree of Heterosis and Increased Reproductive Isolation: A Strategic Design for Breeding Inter-Subspecific Hybrid Rice

**DOI:** 10.1371/journal.pone.0093122

**Published:** 2014-03-25

**Authors:** Zhiwu Dan, Ping Liu, Wenchao Huang, Wei Zhou, Guoxin Yao, Jun Hu, Renshan Zhu, Baorong Lu, Yingguo Zhu

**Affiliations:** 1 State Key Laboratory of Hybrid Rice, College of Life Sciences, Wuhan University, Wuhan, China; 2 Engineering Research Center for Plant Biotechnology and Germplasm Utilization, Ministry of Education, Wuhan University, Wuhan, China; 3 Ministry of Education Key Laboratory for Biodiversity Science and Ecological Engineering, Institute of Biodiversity Science, Fudan University, Shanghai, China; Nanjing Agricultural University, China

## Abstract

The application of heterosis (hybrid vigor) has brought great success to plant breeding, particularly of hybrid rice, achieving significant yield increases. Attempts to explore the heterosis of inter-subspecific hybrids between *indica* and *japonica* rice, which result in even greater yield increases, have greatly increased in the past decades. However, because of the reduced seed setting rate in F_1_ hybrids as a result of increased reproductive isolation, the application of inter-subspecific hybrids in rice has slowed. Understanding the balance between heterosis and the reproductive isolation of inter-subspecific hybrids will facilitate the strategic design of inter-subspecific hybrid breeding. In this study, five *indica* and seven *japonica* rice varieties were chosen as the parental lines of a complete diallel mating design. Data from six group traits from all of the hybrids and inbred lines were collected. We found that the grain weight per plant, grain number per panicle, tiller per plant, thousand grain weight and plant height, which reflected increased heterosis, were associated with the genetic divergence index (GDI) of the parents. Meanwhile, owing to the reduced seed setting rate, which was also associated with the parents' GDI, the grain production of the hybrids was negatively affected. After analyzing the relationships between the GDI of *indica-japonica* parents and the grain weight per plant of the F_1_ hybrids, an ideal GDI value (0.37) for the two *indica-japonica* parents that could provide an optimal balance between the inter-subspecific heterosis and reproductive isolation was proposed. Our findings will help in the strategic design of an inter-subspecific hybrid rice breeding program by identifying the ideal *indica* and *japonica* parents for a hybrid combination to achieve hybrid rice with an optimal yield. This strategic design of an inter-subspecific hybrid rice breeding program will be time saving and cost effective.

## Introduction

Rice is the main staple food for almost half of the world's population. In China, rice constitutes 40% of the total calorie intake [1]. Asian-cultivated rice (*Oryza sativa* L.) consists of two subspecies, *indica* and *japonica*. These subspecies have significantly diverged at the molecular [2]–[5], physiological and biochemical levels [6]–[8]. Regarding morphology and living conditions, *indica* varieties exhibit greater plant heights, longer leaves, and heat and moisture tolerance but are sensitive to low temperatures. These varieties are cultivated at low latitudes and in humid regions. *japonica* varieties have lower plant heights and shorter leaves than do those of *indica* but are tolerant to low temperatures. In addition, these varieties are more suitable for high latitudes and for the lower latitudes of high altitude cultivation. Because of these differences, reproductive isolation emerges in the hybridization between these two subspecies.

Hybrid rice was first widely commercialized in the 1970s in China [9]. Some elite hybrid rice varieties with excellent performances were bred in China, such as Shanyou63 and Luoyou8, which are widely cultivated in Asian countries. However, these varieties are mostly intra-subspecific hybrids, and the heterosis of the hybrids is limited. With the growing global population and decreasing proportion of arable land, increasing rice yield remains an urgent task. The magnitude of heterosis depends on the genetic diversity between the two parents of the hybrids. The greater the genetic difference between the parents, the higher the heterosis [10]. Fortunately, the inter-subspecific hybridization between *indica* and *japonica* can gain powerful heterosis compared with intra-subspecies hybridization [11]–[15]. The utilization of inter-subspecific hybrids is the most feasible approach for realizing super-high yields [1], [13]. Hybrids of *indica* and *japonica* varieties have a yield advantage of approximately 25% [14]. In addition, Dr. G. Khush of the IRRI has also predicted that the combination of IRRI's new plant types with the heterosis of *indica-japonica* may increase the yield potential of tropical rice by 50%.

However, breeders are hampered by the reproductive isolation of inter-subspecies. As a type of evolutionary phenomenon, the F_1_ hybrids often have a low seed setting rate [16], [17]. Even after doubling the number of chromosomes to obtain autotetraploid rice, this problem could still not be resolved [18]. Over recent decades, many studies have investigated the low seed setting rate defect [16], [19]–[25]. Explanations for this phenomenon include pollen sterility, embryonic abortion or incompatibility. Because the interaction of *indica-japonica* hybridization is complex, reproductive isolation is still a major obstacle to breeding inter-subspecific hybrid rice.

Considering that heterosis is determined by the divergence of the parents and that the seed setting rate is controlled by the degree of reproductive isolation in the parental lines, we hypothesized the existence of a critical point at which the conflict between the greater degree of heterosis and the increased reproductive isolation is balanced. At this point, the heterosis of inter-subspecific hybridization can be acquired, and a high seed setting rate can also be realized. In search of this point, twelve rice varieties, five *indica* and seven *japonica*, with different proportions of *indica*-*japonica* content were selected as the mating parents of complete diallel hybridization. An experimental population with 132 hybrids and twelve parental lines was constructed. The genetic divergence index (GDI) of the *indica-japonica* parents and the plant height, grain weight per plant, and yield related trait data were collected. Regression analyses were performed to depict the relationship between the yield and GDI of the parents. After integrating the regression results of the raw phenotypic data, relative low-parent heterosis, relative mid-parent heterosis, and relative better-parent heterosis, an ideal GDI was found and was consistent in reciprocal combinations. This result will help to facilitate the inter-subspecific hybrid rice breeding program and contribute to yield improvement.

## Materials and Methods

A total of 260 rice varieties (CV, the field studies did not include endangered or protected species), which were collected worldwide (China, India, Italy, Japan, Philippines, respectively, and we received the rice samples from fellow researchers, Guangzhu Zhang from Heilongjiang Academy of Agricultural Sciences, Liangming Chen from Nanjing Agricultural University, Liyon Cao from China National Rice Research Institute, Wuhan Zhang from Hunan Hybrid Rice Research Center, Yixuan Lu from Yunnan Academy of Agricultural Sciences, respectively), were planted in the experimental field of the Engineering Research Center for Plant Biotechnology and Germplasm Utilization, Ministry of Education, Wuhan University, in Wuhan (N30° 32′ 22.44″, E114° 22′ 18.21″; no specific permissions were required for the locations in this study) in May 2011. The DNA from every inbred line was extracted using the CTAB method [26]. The proportions of *indica-japonica* content named F_i_ of these inbred lines were determined using the InDel marker estimating method. The selected co-dominant InDel markers have been validated to truly reflect the genetic variation and differentiation of *indica* and *japonica* subspecies[27]. After PCR reactions, electrophoresis, the banding patterns of these rice varieties could be divided into *indica* type which was identical to 93–11 and *japonica* type which was identical to Nipponbare. By calculating the *indica* or *japonica* banding type frequency of all the InDel loci, a total of 12 rice varieties whose F_i_ ranged from 0 to 1 were selected as the parental lines (Table 1). R465 and Balilla, two rice accession resources, were introduced from India and Italy, respectively, by the Chinese government. The remaining species are native to low and high latitudes in China and some are outstanding restorer lines, such as Mianhui725, 610234, and 9311K. All of the selected lines have good agronomic traits and are suitable for breeding programs. The phenotypes of the twelve rice varieties are consistent with the F_i_ value from our previous study. Each of the twelve selected inbred lines were planted in the experimental field of the Hybrid Rice Hainan Experimental Base of Wuhan University in Lingshui (N18° 30′ 22.12″, E110° 2′ 10.72″), Hainan Province, in December 2011. A complete diallel mating design was used to obtain the hybrids. Total of 132 bag hybrids were obtained from March to May 2012.

**Table 1:** The 12 rice varieties used in this study.

Cultivar	F_i_	Origin
R465	1.00	India
Mianhui725	0.92	Sichuan, China
610234	0.88	Hubei, China
9311K	0.82	Sichuan, China
Qianlijing	0.70	Sichuan, China
W1384	0.33	Jiangsu, China
W1383	0.30	Jiangsu, China
W1392	0.20	Jiangsu, China
W1390	0.18	Jiangsu, China
Liaoxing1	0.12	Liaoning, China
Wuyunjing8	0.02	Jiangsu, China
Balilla	0	Italy

The seeds of twelve rice varieties and 132 hybrids were bagged with transparent plasmic bags and buried in water at 28°C for 48 h. These seeds were then transferred to an incubator at a constant temperature of 28°C for 24 h. All of the seeds were planted in soil in the experimental field of the Hybrid Rice Ezhou Experimental Base of Wuhan University in Ezhou (N30° 22′ 19.82″, E114° 44′ 59.17″), Hubei Province, on May 13, 2012 and were transplanted on June 10, 2012. A randomized block design with three replications was applied. A total of 10 individual plants of each replicate were planted at a spacing of 16.5×26.4 cm. Four plants from a cytoplasmic male-sterile line named YTA were located around these individuals to minimize the marginal effect. The field managements were applied as recommended. Due to low germination, the seedling numbers of some combinations were not sufficient for statistical analyses, and therefore, 122 hybrid combinations remained. Five plants in the middle of each repeat were chosen to collect the field data. All of the parental lines and the hybrid plant height (from the surface of the soil to the highest part of the plant) and tiller number were measured during the maturation stage. The grain weight per plant (GWP) was weighed. The grain number per plant and the empty grain number were measured using a Seed Counting Machine (PME-1 Seed Auto-counting Machine, Shanke Equipment, Shanghai, China). The grain number per plant divided by the tiller (tillers with grain) number per plant (TPP) determined the grain number per panicle (GNP). The seed setting rate (SSR) was calculated by dividing the full grain number per plant by the total grain number per plant. Means over replications were calculated for each trait and used in the data analysis.

The GDI of the parental lines per hybrid was calculated as GDI  =  |F_ia_ − F_ib_|, where F_ia_ is parent a′s F_i_, and F_ib_ is parent b′s F_i_. The relative low-parent heterosis (LPH) was determined using the equation *LPH  =  (F_1_ − P_low_)/P_low_*. The relative mid-parent heterosis (MPH) was determined using the equation *MPH*  =  (*F_1_* − *MP)/MP*. The relative better-parent heterosis (BPH) was determined using the equation *BPH  =  (F_1_ − P_high_)/P_high_*. *F_1_* represents the hybrid data; *P_low_* represents the lower data of the parent. *MP* represents the means of the parent. *P_high_* represents the higher data of the parent. All of the trait data analyses were performed using the software SPSS 19.0 (SPSS, Chicago, Illinois, USA).

## Results

### *Indica*-*japonica* inter-subspecific hybridization acquired heterosis in yield, yield-related agronomic traits and plant height

**Figure 1:**
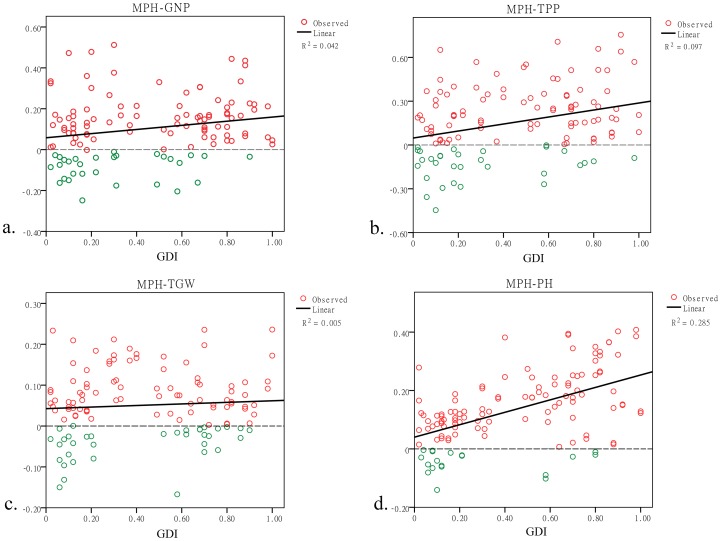
Relative mid-parent heterosis in yield-related traits and plant height. MPH indicates relative mid-parent heterosis. The solid line represents the total changing trend. The dashed line represents the critical value 0. The red circles represent hybrids that show hybrid vigor, and the yellow circles represent hybrids that show hybrid weakness. a, b, c, and d represent linear regressions between the GDI and the relative mid-parent heterosis of grain number per panicle, tiller per plant, thousand grain weight and plant height, respectively.

Each of the five traits exhibited heterosis (Table 2). The grain weight per plant has the strongest relative mid-parent heterosis, which is approximately 24%, consistent with previous reports [14]. Regarding the remaining three yield-related traits, the highest average value of the relative mid-parent heterosis was the tiller number per plant, which was as high as 15.38%. The lowest was the thousand grain weight of 5.10%, and the grain number per panicle was 10.29%. The magnitudes of heterosis in these three traits were all smaller than the grain weight per plant. Considering that the yield is a complex trait, these results may indicate that the heterosis of yield may be a cumulative outcome of yield-related traits. Furthermore, as a typical nutritional trait, the relative mid-parent heterosis of plant height was as high as 13.45%. The highest percentage of the hybrids displaying positive relative mid-parent heterosis (MPH≥0) was 84.68% in plant height. The remaining three yield-related traits were greater than 70%, and the grain weight per plant was 68.03%. These results demonstrate that heterosis is acquired through inter-subspecific hybridization both in reproductive traits and nutritional trait. We then analyzed the relationships between the GDI and the relative mid-parent heterosis of grain number per plant, tiller per plant, thousand grain weight and plant height (Figure 1). Except for the thousand grain weight, the remaining three traits showed a significant increasing trend of heterosis, demonstrating that the larger the genetic divergence, the higher the degree of heterosis. Therefore, the application of *indica-japonica* inter-subspecific hybridization seems to be a practical method to improve the rice yield.

**Table 2:** MPH in yield, yield-related traits and plant height.

Phenotype	No.	Average % MPH	%MPH values^a^
Grain weight per plant	122	23.96%	68.03%
Grain number per panicle	122	10.29%	74.59%
Tiller per plant	122	15.38%	74.59%
Thousand grain weight	122	5.10%	72.13%
Plant height	124	13.45%	84.68%

a The % MPH values refer to the percent of hybrids that exhibit positive relative mid-parent heterosis.

### The seed setting rate was closely associated with the GDI of *indica*-*japonica* parental lines

**Figure 2:**
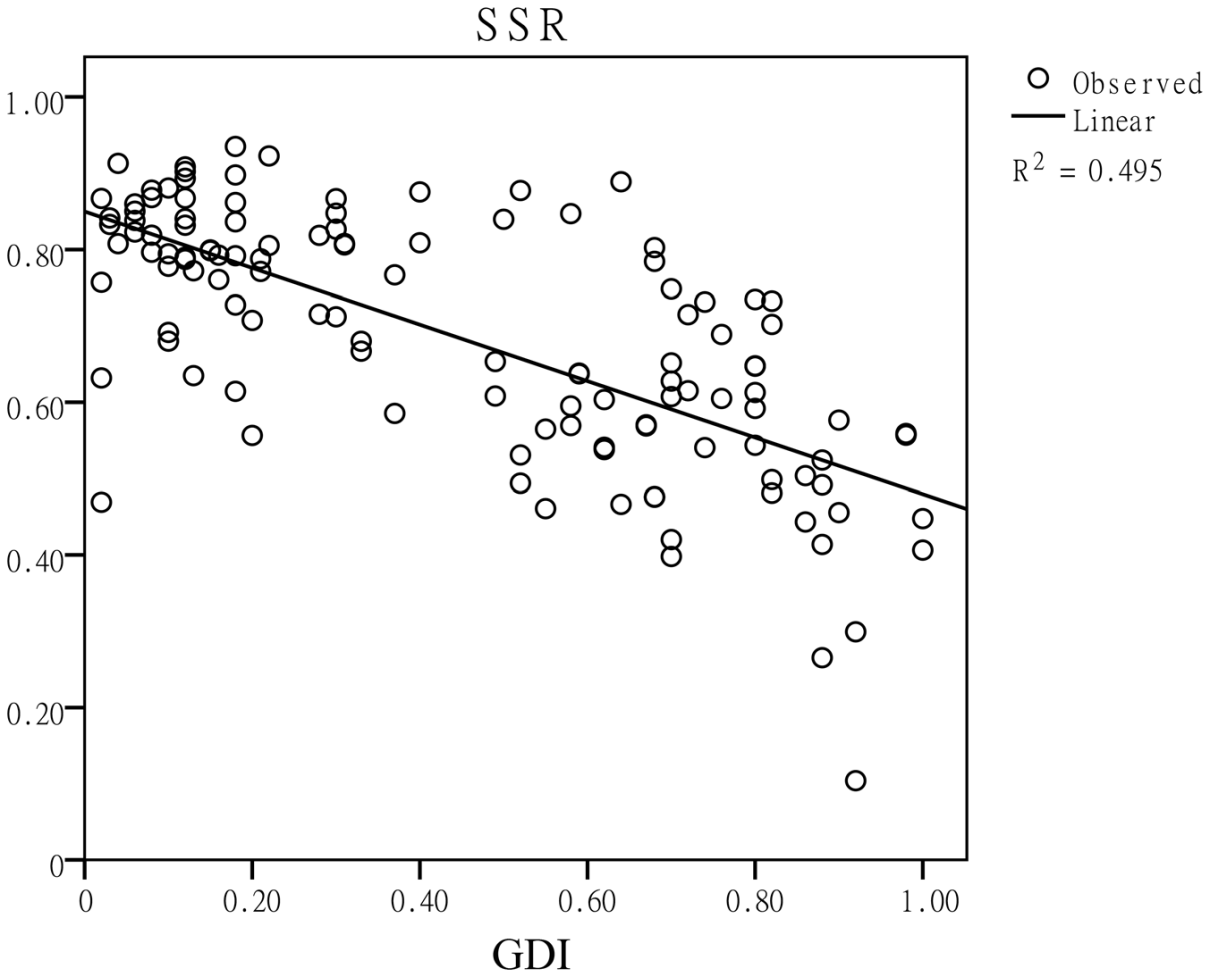
Linear regression between the seed setting rate and the GDI. SSR and GDI represent the seed setting rate and genetic divergence index, respectively.

**Figure 3:**
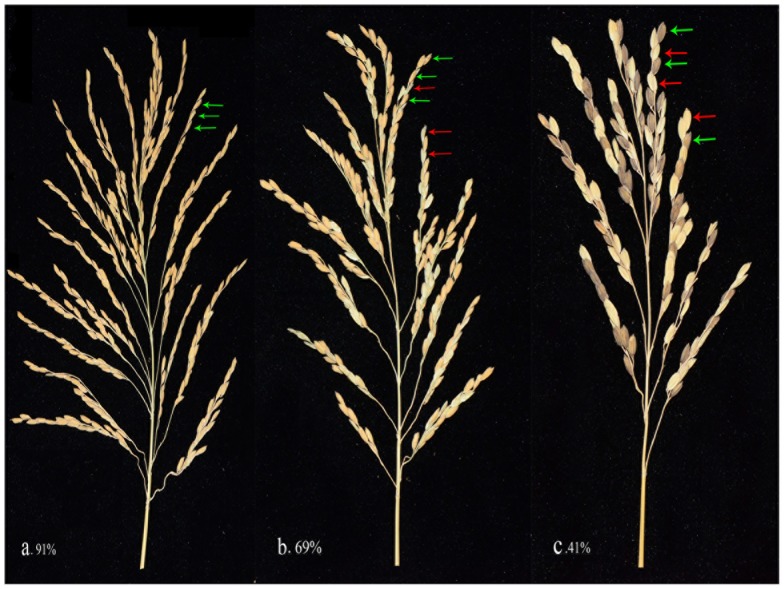
Panicles of hybrids with different GDI values. a. Qianlijing/9311k, GDI = 0.12, SSR = 91%; b. 610234/Liaoxing1, GDI = 0.76, SSR = 69%; c. R465/Balilla, GDI = 1, SSR = 41%. The green arrow indicates full grains; the red arrow indicates empty grains.

The *indica* and *japonica* varieties are two subspecies of Asian-cultivated rice. Through evolution, reproductive isolation came to exist in their hybrid offspring. In this study, when the GDI increased, indicating that the degree of reproductive isolation between the parents became more serious, their hybrids' seed setting rate decreased dramatically (Figure 2). Figure 3 shows the changes in the seed setting rate as the GDI increased. When the parents were closely related, the seed setting rates were greater than 80% (Figure 3a). If the GDI was close to 0.5, the seed setting rate decreased to nearly 60%, which could be regarded as semi-sterile. In addition, the seed setting rate could reach approximately 40% when the two parents were typical *indica* and *japonica* varieties (Figure 3c). The lowest seed setting rate in this study was 10% in the Mianhui725/Balilla combination. Additionally, in the linear regression model, the seed setting rate can be predicted using the equation SSR = 0.85−0.37*GDI.

### The grain weight per plant decreased significantly when the seed setting rate decreased

**Figure 4:**
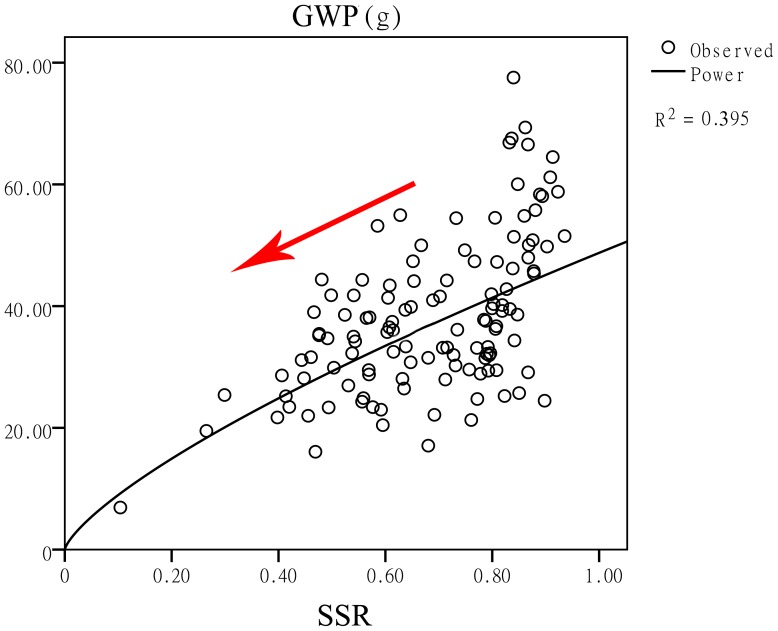
Linear regression between the grain weight per plant and the seed setting rate. SSR and GWP represent the seed setting rate and grain weight per plant, respectively.

The seed setting rate is one of the most important factors in determining the grain weight per plant. A low seed setting rate of inter-subspecific hybridization has puzzled rice breeders for many years. Figure 4 depicts the relationships between the grain weight per plant and the seed setting rate. Hybrids with a high seed setting rate could achieve a high grain yield value. At a seed setting rate value of approximately 85%, the grain weight per plant peaked at approximately 80 g/plant. However, this example may be extreme in the population, and there were still some hybrids with low yield performance at a seed setting rate of 85%. Subsequently, when the seed setting rate reached 90%, the grain weight per plant was quite excellent. No combinations remained under the regression curve. Once the seed setting rate is high enough, the yield will be improved. Therefore, although the yield is a complex trait that is influenced by many agronomic traits, the seed setting rate is a key element in determining the final output. The relationship between these two traits was GWP = 0.735*ln(SSR)+48.751.

### Linear regression between the grain weight per plant and the GDI

**Figure 5:**
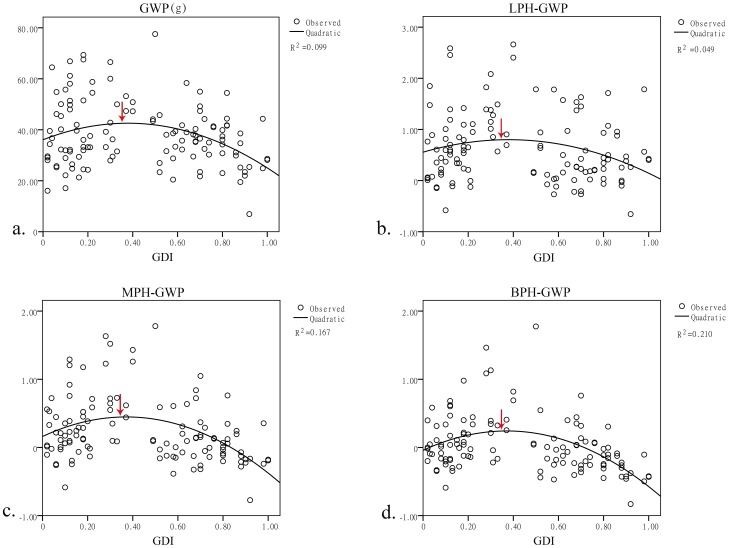
Regression model between GWP, LPH-GWP, MPH-GWP, BPH-GWP and GDI. a. Quadratic regression between the GDI and the GWP; the peak is at GDI = 0.3783. b. Quadratic regression between the GDI and the LPH-GWP; the peak is at GDI = 0.3792. c. Quadratic regression between the GDI and the MPH-GWP; the peak is at GDI = 0.3712. d. Quadratic regression between the GDI and the BPH-GWP; the peak is at GDI = 0.3713.

**Figure 6:**
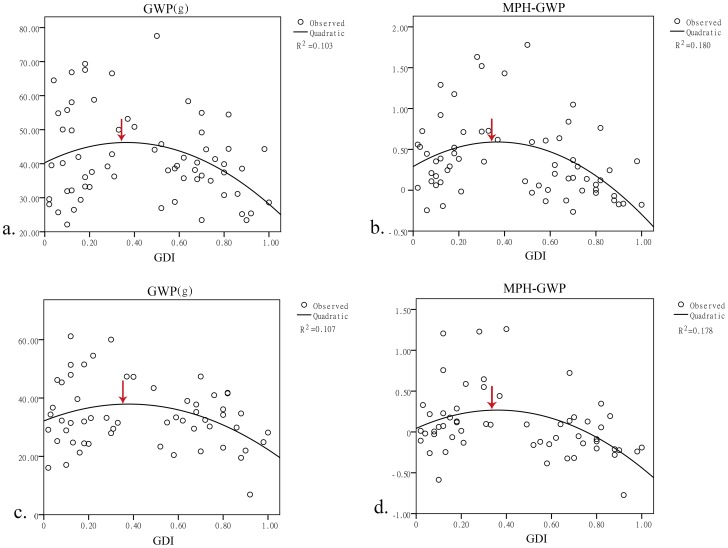
Linear regression between the GWP and the MPH-GWP of reciprocal combinations and the GDI. a. Quadratic regression between the GDI and the GWP of group 1; the peak is at GDI = 0.3645. b. Quadratic regression between the GDI and the MPH-GWP of group 1; the peak is at GDI = 0.378. c. Quadratic regression between the GDI and the GWP of group 2; the peak is at GDI = 0.3681. d. Quadratic regression between the GDI and the MPH-GWP of group 2; the peak is at GDI = 0.363.

Because the seed setting rate was negatively correlated with the GDI, it was positively correlated with the grain weight per plant; therefore, we analyzed the relationship between the GDI and the grain weight per plant. The correlation results showed that the grain weight per plant and the GDI were significant at the 0.05 level (Table 3). A regression model was subsequently constructed to further investigate the relationship between the grain weight per plant and the GDI (Figure 5a). The regression equation was GWP = 36.08+34.294*GDI-45.342*GDI^2^, which is a quadratic function. The highest point was at GDI = 0.3783, indicating that the ideal GDI of the parents may be 0.3783. Then, the relationships between the GDI and the LPH, MPH, and BPH were analyzed (Figure 5b, c, d). It was surprising that all of these quadratic regression outcomes had approximately the same peak, at 0.37. We thus concluded that when the GDI of the parental lines is 0.37, the hybrids will have the highest probability of obtaining the best yield performance. Considering that cytoplasmic differences may influence the hybrids, this value may not be suitable for traditional breeding applications. We divided the total experimental population into two groups that were reciprocal combinations. Among these reciprocal combinations, those with higher values were classified into group 1, and those with lower values were in group 2. The same regression analyses were performed (Figure 6). The ideal GDI was close to 0.37, indicating that this value is consistent in different cytoplasm with the same nuclear background.

**Table 3:** Correlations between the GDI and the GWP, the relative heterosis of the GWP.

		GWP(g)	LPH-GWP	MPH-GWP	BPH-GWP
GDI	Pearson Correlation	−0.198*	−0.138	−0.269**	−0.301**
	Sig. (2-tailed)	0.029	0.131	0.003	0.001
	N	122	122	122	122

**. Correlation is significant at the 0.01 level (2-tailed).

*. Correlation is significant at the 0.05 level (2-tailed).

## Discussion

Experiments investigating the relationship between the parental lines and the hybrid performance were conducted over recent decades in order to make use of the heterosis of inter-subspecific hybrids [15], [28]–[32]. However, no practical theoretical method has been proposed to guide rice breeders for correctly selecting restorer lines for a sterile line. Currently, large amounts of traditional testing hybridizations are still being used, which are time- and energy-consuming.

### Higher degree of heterosis from *indica-japonica* inter-subspecific hybridization

Our results showed that the relative mid-parent heterosis of the grain weight per plant can be as high as approximately 24%, which is close to 25% [14]. This result may reflect the cumulative effects of heterosis on yield-related traits. The grain number per panicle, tiller per plant and thousand grain weight displayed increasing trends to some extent. The larger genetic distance, the higher the degree of heterosis. Inter-subspecific hybridization of rice will make a powerful contribution to solving the problems of a growing global population and decreasing arable land and meeting the demands of an environmentally friendly, low carbon output.

### The importance of choosing an appropriate estimating method to determine the GDI of *indica-japonica* parental varieties

Knowing that reproductive isolation is caused by the genetic divergence between parents, many studies have estimated the degree of differentiation between the *indica-japonica* rice varieties [4], [33]–[36], and efforts have also been made to determine the relationship between genetic divergence and heterosis [37]–[39]. Most reports correctly concluded that the larger the genetic distance between parents, the stronger the heterosis of the hybrids. However, in regard to the relationships between the yield and heterosis, people found it incomprehensible and obtained different and even contradictory outcomes. The reason for these discrepancies may be that the DNA markers chosen to classify the genetic distance of the parents were not suitable. Because there are so many different loci between the sequences of *indica* and *japonica*, these differences may not show the true genetic distance. Therefore, selecting an appropriate estimating tool is quite important.

Speciation is connected to reproductive isolation. In inter-subspecific hybridizations, the seed setting rate of the hybrids should be a favorable indicator for the real genetic divergence of the parents. Therefore, the correlations between the genetic divergence and the seed setting rate must be verified to be true. In this study, when the GDI increased, the seed setting rate decreased synchronously (Figure 2, Figure 3), indicating that those InDel markers are feasible for and effective at estimating the genetic divergence.

### Balance between a higher degree of heterosis and increased reproductive isolation in the inter-subspecific hybridization to achieve a maximum yield

The seed setting rate is related to the reproductive isolation of inter-subspecies caused by speciation, and the speciation procedure also introduces heterosis. Therefore, we cannot ignore reproductive isolation in exploiting heterosis. However, we can find a balance between these two aspects. All of the linear regression results were optimal at GDI = 0.37, and this value remained constant in the reciprocal combinations. At this point, the heterosis of inter-subspecies has been acquired, and reproductive isolation can be avoided. This result provides a theoretical guide to *indica-japonica* hybrid rice breeding. Rice breeders can evaluate the degree of *indica-japonica* differentiation (F_i_) of the restorer lines and identify suitable *indica* and/or *japonica* sterile lines based on the GDI value of 0.37 to achieve hybrid rice combinations with an optimal yield. The strategic design for the breeding program of inter-subspecific hybrid rice will be time-saving and cost-effective.

## Supporting Information

File S1 **Phenotypic data.**
(XLSX)

## References

[1] Cheng S, Zhuang J, Fan Y, Du J, Cao L (2007) Progress in research and development on hybrid rice: a super-domesticate in China. Annals of Botany 100: 959–966. 17704538 10.1093/aob/mcm121 PMC2759200

[2] Zhang Q, Saghai Maroof MA, Lu TY, Shen BZ (1992) Genetic diversity and differentiation of *indica* and *japonica* rice detected by RFLP analysis. Theor Appl Genet 83: 495–499. 24202597 10.1007/BF00226539

[3] Yang GP, Saghai Maroof MA, Xu CG, Zhang Q, Biyashev RM (1994) Comparative analysis of microsatellite DNA polymorphism in landraces and cultivars of rice. Mol Gen Genet 245: 187–194. 7816026 10.1007/BF00283266

[4] Garris AJ, Tai TH, Coburn J, Kresovich S, McCouch S (2005) Genetic structure and diversity in *Oryza sativa* L. Genetics 169: 1631–1638. 15654106 10.1534/genetics.104.035642 PMC1449546

[5] Johns MA, Mao L (2007) Differentiation of the two rice subspecies *indica* and *japonica*: a Gene Ontology perspective. Funct Integr Genomics 7: 135–151. 17131138 10.1007/s10142-006-0036-1

[6] Glaszmann JC (1987) Isozymes and classification of Asian rice varieties. Theor Appl Genet 74: 21–30. 24241451 10.1007/BF00290078

[7] Khush GS (1997) Origin, dispersal, cultivation and variation of rice. Plant Molecular Biology 35: 25–34. 9291957

[8] Nakamura Y, Sakurai A, Inaba Y, Kimura K, Iwasawa N, et al (2002) The fine structure of amylopectin in endosperm from Asian cultivated rice can be largely classified into two classes. Starch/Stärke 54: 117–131.

[9] Lin S, Yuan L (1980) Hybrid rice breeding in China. The International Rice Research Institute, LOS, BANOS, LAGUNA, PHILIPPINES, PO BOX, 933, MANILA, PHILIPPINES: 35–52.

[10] Khush GS, Peng S (1996) Breaking the Yield Frontier of Rice. Reynolds MP, Rajaram SP and McNab A, eds 1996 Increasing Yield Potential in Wheat: Breaking the Barriers Mexico, D.F.: CIMMYT: 36–51.

[11] Jiang J, Li J, Xu Z, Zhang L, Jin C (2002) Study on heterosis of hybridization between *indica* and *japonica* rice I.The heterosis of main morphoiogy characters and fibrovascuiar bundies characters. Journal of Jilin Agricultural Sciences 27: 3–7.

[12] Jiang J, Li J, Xu Z, Zhang L, Jin C (2002) Studies on the heterosis of hybridization between *indica* and *japonica* rice II. The heterosis of main physiology characters and economic characters. Journal of Jilin Agricultural Sciences 27: 3–6.

[13] Yuan L (1998) Hybrid rice breeding for super high yield. Denning GL, Mew TW, editors. 1998. China and IRRI: Improving China's rice productivity in the 21st century Manila (Philippines): International Rice Research Institute. 104 p: 10–12.

[14] Khush GS (1995) Breaking the yield frontier of rice. GeoJournal 35: 329–332.

[15] Zeng S, Yang X, Lu Z (1980) The heterosis of F_1_ hybrids between *Oryza Sativa* subsp. *hsien* and O. Sativa subsp. *keng* . Acta Agronomica Sinica 6: 193–202.

[16] Li Z, Pinson Shannon RM, Paterson AH, Park WD, Stansel JW (1997) Genetics of hybrid sterility and hybrid breakdown in an intersubspecific rice(*Oryza sativa* L.) population. Genetics 145: 1139–1148. 9093864 10.1093/genetics/145.4.1139 PMC1207882

[17] Ouyang Y, Liu Y, Zhang Q (2010) Hybrid sterility in plant: stories from rice. Plant Biology 13: 186–192. 10.1016/j.pbi.2010.01.002 20153244

[18] He JH, Shahid MQ, Li YJ, Guo HB, Cheng XA, et al (2011) Allelic interaction of F_1_ pollen sterility loci and abnormal chromosome behaviour caused pollen sterility in intersubspecific autotetraploid rice hybrids. Journal of Experimental Botany 62: 4433–4445. 21624978 10.1093/jxb/err098 PMC3170543

[19] Song X, Qiu SQ, Xu CG, Li XH, Zhang Q (2005) Genetic dissection of embryo sac fertility, pollen fertility, and their contributions to spikelet fertility of intersubspecific hybrids in rice. Theor Appl Genet 110: 205–211. 15672255 10.1007/s00122-004-1798-2

[20] Zhang H, Tan G, Yang L, Yang J, Zhang J, et al (2009) Hormones in the grains and roots in relation to post-anthesis development of inferior and superior spikelets in *japonica/indica* hybrid rice. Plant Physiology and Biochemistry 47: 195–204. 19117763 10.1016/j.plaphy.2008.11.012

[21] Li W, Zeng R, Zhang Z, Ding X, Zhang G (2008) Identification and fine mapping of *S-d*, a new locus conferring the partial pollen sterility of intersubspecific F_1_ hybrids in rice (*Oryza sativa* L.). Theor Appl Genet 116: 915–922. 18274725 10.1007/s00122-008-0723-5

[22] Singh SP, Sundaram RM, Biradar SK, Ahmed MI, Viraktamath BC, et al (2006) Identification of simple sequence repeat markers for utilizing wide-compatibility genes in inter-subspecific hybrids in rice (*Oryza sativa* L.). Theor Appl Genet 113: 509–517. 16788798 10.1007/s00122-006-0316-0

[23] Li H, Zhou S, Huang D, Lu D, Lai S, et al. (2012) Research progress on *indica/japonica* rice cross breeding. Guangdong Agricultural Sciences 21..

[24] Mizuta Y, Harushima Y, Kurata N (2010) Rice pollen hybrid incompatibility caused by reciprocal gene loss of duplicated genes. PNAS 107: 20417–20422. 21048083 10.1073/pnas.1003124107 PMC2996679

[25] Ji Q, Lu J, Chao Q, Zhang Y, Zhang M, et al (2010) Two sequence alterations, a 136 bp InDel and an A/C polymorphic site, in the *S5* locus are associated with spikelet fertility of *indica-japonica* hybrid in rice. Journal of Genetics and Genomics 37: 57–68. 20171578 10.1016/S1673-8527(09)60025-4

[26] Murray MG, Thompson WF (1980) Rapid isolation of high molecular weight plant DNA. Nucleic Acids Research 8: 4321–4325. 7433111 10.1093/nar/8.19.4321 PMC324241

[27] Lu B, Cai X, Jin X (2009) Efficient *indica* and *japonica* rice identification based on the InDel molecular method:its implication in rice breeding and evolutionary research. Progress in Natural Science: 1241–1252.

[28] Sun J, Tang L, Zhu Q, Xue Y, Cai Y (1999) Progress of study on ultilization of F_1_ heterosis between *indica* and *japonica* rice subspecies I. Character relationships between F_1_ of *indica*–*japonica* and their parents. Journal of Tianjin Agricultural College 6: 1–7.

[29] Sun C, Tingbo J, Chen L, Wu C, Li Z, et al (2000) Studies on the relationship between heterosis and genetic differentiation in hybrid rice (*Oryza sativa* L.). Acta Agronomica Sinica 26: 641–649.

[30] Li R, Xu CG, Yang ZY, Wang XK (1998) The extent of parental genotypic divergence determines maximal heterosis by increasing fertility in intersubspecific hybrids of rice (*Oryza sativa* L.). Molecular Breeding 4: 205–214.

[31] Yang S, Shen X, Gu W, Cao D (1962) Research on *indica-japonica* hybridization breeding. Zuowuxuebao 1: 97–102.

[32] Xu H, Tao S, Tang L, Zhang W, Zhao M, et al (2012) Research progress of diferentiation and hybrid breeding between *indica* and *japonica* rice. Journal of Shenyang Agricultural University 43: 704–710.

[33] Qi Y, Zhang H, Zhang D, Wang M, Sun J, et al (2009) Assessing *indica-japonica* differentiation of improved rice varieties using microsatellite markers. Journal of Genetics and Genomics 36: 305–312. 19447379 10.1016/S1673-8527(08)60119-8

[34] Wang M, Zhu Z, Tan L, Liu F, Fu Y, et al (2013) Complexity of *indica-japonica* varietal differentiation in Bangladesh rice landraces revealed by microsatellite markers. Breeding Science 63: 227–232. 23853518 10.1270/jsbbs.63.227 PMC3688385

[35] Vanniarajan C, Vinod KK, Pereira A (2012) Molecular evaluation of genetic diversity and association studies in rice(*Oryza sativa* L.). Journal of Genetics 91: 9–19. 22546822 10.1007/s12041-012-0146-6

[36] Jiang T, Sun C, Li R, Li Z, Wang X (1999) Study on classification of crosses and their parents by RFLP markers in two-line system hybrid rice. Scientia Agricultura Sinica 32: 1–10.

[37] Jiang T, Li R, Sun C, Li Z, Wang X (1999) A study on the relation of combining ability in main traits of two–line system hybrid rice and parental *indica-japonica* diferentiatin. Journal of Agricultural Biotechnology 7: 295–300.

[38] Long H, Xu M, Zhang S (2003) A preliminary study on the relationship between the *indica-japonica* differentiation of parents and heterosis in Dian type hybrid rice by RAPD markers. Scientia Agricultura Sinica 36: 1–6.

[39] Li YL, Ma JW, Yang XX, Xu MH, Zhao FP (2012) Analysis of relationship between *indica-japonica* differentiation of parents and heterosis in Dian type hybrid rice by SSR markers. Southwest China Journal of Agricultural Science 25: 347–353.

